# Is a Blanket Elective Single Embryo Transfer Policy Defensible?

**DOI:** 10.5041/RMMJ.10299

**Published:** 2017-04-28

**Authors:** Eli Y. Adashi, Norbert Gleicher

**Affiliations:** 1 Professor of Medical Science, The Warren Alpert Medical School, Brown University, Providence, RI, USA; 2 Medical Director and Chief Scientist, The Center for Human Reproduction, New York, NY, USA; 3 President, The Foundation for Reproductive Medicine, New York, NY, USA; 4 Professor (Adj.), Stem Cell Biology and Molecular Embryology Laboratory, Rockefeller University, New York, NY, USA; 5 Professor (Adj.), Department of Obstetrics and Gynecology, Vienna University School of Medicine, Vienna, Austria

**Keywords:** Double embryo transfer, *in vitro* fertilization, multiple pregnancy, single embryo transfer

## Abstract

For the purpose of reducing maternal and neonatal morbidity, elective single transfer (eSET) in *in vitro* fertilization (IVF) was first proposed in 1999. The purpose of this review is to summarize recent oral debate between a proponent and an opponent of expanded eSET utilization in an attempt to determine whether a blanket eSET policy, as is increasingly considered, is defensible. While eSET is preferable when possible, and agreed upon by provider and patient, selective double embryo transfer (DET) must be seriously entertained if deemed more appropriate or is desired by the patient. Patient autonomy, let alone prolonged infertility and advancing age, demand nothing less. Importantly, IVF-generated twins represent only 15.7% of the national twin birth rate in the United States. Non-IVF fertility treatments have been identified as the main cause of all multiple births for quite some time. However, educational and regulatory efforts over the last decade, paradoxically, have exclusively only been directed at the practice of IVF, although IVF patient populations are rapidly aging. It is difficult to understand why non-IVF fertility treatments, usually applied to younger women, have so far escaped attention. This debate on eSET utilization in association with IVF may contribute to a redirection of priorities.

## INTRODUCTION

Elective single embryo transfer (eSET) was first proposed in 1999 by Finnish investigators with the argument that twin pregnancies increase maternal and neonatal risks to offspring in association with fertility treatments and should thus be avoided.^1^ The clinical utilization of eSET has increased ever since, first in Europe^2^ and Canada,^3^ and more recently also in the US,^4^ with even the national professional organization expressing support for the concept.^5^

In the US the subject also attracted the attention of the March of Dimes, likely the nation’s pre-eminent organization dedicated to the eradication of premature births, which in 2013 engaged the Hastings Center, an independent bioethics research institute, to investigate the subject and issue a report. For unclear reasons, a full-length report has yet to be published, though an abbreviated summary appeared in *Fertility Sterility* in 2014 strongly encouraging expanded use of eSET.^6^ Additional publications saw press in support of this policy.^7^,^8^ A preliminary draft of the final report, circulated among members of an advisory expert panel the Hastings Center had gathered in a two-day symposium, offered, however, a more subtle assessment, and was more reflective of some opinions opposed to the expanded utilization of eSET.^7^

The purpose of this manuscript is to recapitulate a recent oral debate between a proponent and an opponent of expanded eSET utilization in an attempt to determine whether a blanket eSET policy, as is increasingly considered, is defensible.^9^ E.Y.A. in these pages represents the pro-eSET view, while N.G. argues against the eSET position. The debate in question took place at the *Annual 2016 Conference of The Foundation for Reproductive Medicine on Translational Reproductive Biology and Clinical Reproductive Endocrinology* on November 19, 2016 in New York City.

## YES: TWINS CONSTITUTE AN ADVERSE OUTCOME OF IVF

Since the basic argument in support of eSET is increased maternal and neonatal risk, a basic question of this debate has to be whether or not twins truly, as has been suggested, constitute an adverse *in vitro* fertilization (IVF) outcome.^1^ First raised by Gleicher and associates a decade ago, this question is an important one. In their initial paper, “The Relative Myth of Elective Single Embryo Transfer,” published in 2006 in *Human Reproduction*, and in several subsequent contributions, Gleicher and associates laid out the case against a blanket eSET policy.^10^–^13^ The latest contribution to this series saw press in 2016.^14^

No other investigators so far appear to have challenged in print the notion that twin pregnancies must be avoided at all cost by the universal deployment of eSET. Gleicher et al., thus, added important nuance to the eSET versus double embryo transfer (DET) debate.

E.Y.A. may well lack the qualifications to serve as a representative of the pro-eSET view, since he never espoused a panoptic eSET policy or has ruled out the prospect of a selective discretionary DET option, absent medical contraindications, when desired by a patient. In other words, E.Y.A. holds the opinion that eSET is preferable when possible, and agreed upon by provider and patient, and that selective DET must be seriously entertained if deemed more appropriate or is desired by the patient. Patient autonomy, let alone prolonged infertility and advancing age, demand nothing less.

It also appears worth asking whether the United States, in fact, represents a fitting environment for a blanket eSET policy. We would argue that, likely, the answer is “no”: It currently does not yet (as some other countries) pursue an almost blanket eSET policy. The latest published guidelines of the American Society for Reproductive Medicine (ASRM) in patients with favorable prognosis call for eSET (at blastocyst stage) in patients 35 and younger, and for DET for patients in the 35–37 and 38–40 ranges. Double embryo transfers are also recommended for all patients who are 37 and younger and do not have a favorable prognosis.^5^ Further updated guidelines are pending and can be expected to expand recommendations for eSET utilization. For now at least, flexibility still reigns supreme.

If whether or not twin pregnancies constitute an undesirable outcome of IVF were to be the sole question, the answer would be simple: Following IVF or natural conception, twin pregnancies are, indeed, best avoided.^15^–^19^ Ours, after all, is a mono-ovulatory uniparous species that is ill-suited to concurrent multiple progeny.^20^ Instead, the debate should be about the acceptability of twin pregnancies in the context of a discretionary DET policy, which is to be applied if and when eligible patients wish to or, perhaps, even should pursue this route. This debate should also be about the defensibility of a rigid blanket eSET policy, as distinct from a flexible patient-centered policy that makes room for a discretionary DET paradigm. Inevitably, in this context, the maternal and neonatal risks associated with a twin pregnancy must not be compared with those of an isolated singleton pregnancy but rather with those of two consecutive ones (to be further discussed below).

The case against a blanket eSET policy, as framed by Gleicher and colleagues, argues that any blanket eSET policy is: (1) paternalistic towards DET-eligible subjects pressed to conceive by prolonged infertility and/or age; (2) unethical towards DET-eligible subjects in whom successive eSETs could delay or preclude conception; (3) disadvantageous to DET-eligible subjects whose live birth rates may decline; (4) inconsiderate of DET-eligible subjects in whom successive eSETs could raise costs and efforts; and (5) incapable of assuring a risk level lower than that displayed by twin pregnancies.

E.Y.A. is in in complete agreement with the first two arguments framed by Gleicher and associates, namely, that a blanket eSET policy is paternalistic and unethical when applied to DET-eligible subjects. Such patients would include but need not be limited to those who in the absence of medical contraindications prefer an accelerated approach to family building. Inevitably, such patients must be deemed prepared to assume maternal and neonatal risks that have been associated with a multiple gestation. In general, such patients are older and/or afflicted with age-inappropriate ovarian function. What is more, such patients may also have previously experienced IVF failures. Finally, it is not beyond the realm of possibility that some of the patients in question may lack the means to embark on what could prove to be a long stretch of uncertainty. Such patients deserve to proceed with DET.

E.Y.A. is similarly in general agreement with the third argument of Gleicher and associates to the effect that a blanket eSET policy is disadvantageous to DET-eligible subjects whose live birth rates may decline. Indeed, analyzed in isolation, the success rates of DETs are superior to those of eSET counterparts.^21^–^23^ For now at least, this conclusion appears unassailable. That said, in the context of young good-prognosis patients, the cumulative live birth rate of two sequential eSETs is comparable if not superior to that of a single DET.^24^,^25^ Such outcomes, however, come at a price; that is, they require successive eSET cycles, potential conception delays, and a modicum of uncertainty. To some, the price of entry may be acceptable. Others, however, would not hear of it, instead choosing to pursue the DET route.

With respect to the fourth argument of Gleicher and colleagues, E.Y.A. generally concurs with the notion that a blanket eSET policy is inconsiderate of DET-eligible subjects in whom successive eSETs could raise the level of effort. Less certainty exists, however, as to whether or not eSET is costlier than DET, though the truism of such an assumption strikes one as intuitive. The relative costs associated with DET versus two sequential eSETs have, however, yet to be thoroughly studied. At the very least, placing a question mark by this element of the debate appears to be in order.

A 2007 contribution by Fiddelers et al. concluded that “DET is the most expensive strategy.” Elective single embryo transfer (eSET) “is [however] only preferred from a cost-effectiveness point of view when performed in good prognosis patients and when frozen/thawed cycles are included.”^24^ A 2015 contribution by Crawford et al. stated that “the estimated total ART [assisted reproductive technology] treatment and pregnancy/infant-associated medical costs were $580.9 million for 10,000 DETs started in 2012. If performed as sequential single ETs [embryo transfers], estimated costs would have decreased by $195.0 million to $386.0 million.”^25^ Clearly more work could and should be done relative to these comparative cost estimates, especially since they do not include lifelong earnings of newborns. It appears premature at this time to rely on cost considerations to support or oppose the alternative approaches under discussion in this paper.

Finally, addressing the fifth argument of Gleicher and colleagues, E.Y.A. remains unconvinced that a blanket eSET policy is incapable of assuring lower maternal and neonatal risk levels than displayed by twin pregnancies. At least two published retrospective studies did in fact pursue such comparisons. In 2013, Sazonova et al. cross-linked data from IVF clinics with the Swedish Medical Birth Registry to retrieve obstetrical outcomes.^26^ The authors concluded that “the neonatal and maternal outcomes were *dramatically* better for women undergoing two IVF singleton pregnancies compared with one IVF twin pregnancy after double-embryo transfer.” Increased maternal risks included preterm premature rupture of membranes (PPROM), pre-eclampsia, and need for Cesarean section, but not placenta previa, placental abruption, or gestational diabetes. Increased neonatal risks included preterm birth, small for gestational age, respiratory complications, sepsis, and jaundice, but not perinatal mortality, below 7 Apgar scores, congenital anomalies, or mortality in the first year of life.^26^

In a more recent paper, La Sala et al. also concluded that “the overall risk of perinatal complications was significantly higher in patients who had one twin delivery rather than patients who had two consecutive singleton deliveries.”^27^ However, no difference between groups was detected for intrauterine fetal demise, neonatal death, perinatal mortality, and neonatal intensive care unit (NICU) admission. Taken together, these two papers suggest that additional work is required to clarify this element of the debate. Here again, it may be premature at this time to rely on maternal and neonatal risk data to support or oppose the alternative approaches under discussion today.

On December 23, 2015, the US Division of Vital Statistics of the National Centers for Health Statistics reported that the 2014 national twin birth rate reached an all-time high of 33.9 per 1,000 live births.^28^ The adverse consequences of a rising national twin birth rate cannot be overstated. Notably, 11% and 59%, respectively, were born “very preterm” (under 32 weeks) and “preterm” (under 37 weeks). What is more, 10% and 55% of twins in question, respectively, were characterized as “very low birthweight” (<1,500 g) and “low birthweight” (<2,500 g). In a word, more than one of every two twins born in 2014 in the United States was either preterm or low birthweight. A total of 18 states registered twin birth rates in excess of the overall national figure.^28^ As recently as 2014, the last year for which reliable data are available, the contribution of IVF accounted, however, for only 15.7% of the national twin birth rate.^23^ As such, the contribution of IVF to the national twin birth rate has remained largely unchanged for at least the last decade. Improved as the circumstance may appear, there clearly is room for further improvement, not to mention a great need to address the contribution of non-IVF fertility-promoting technologies.^29^,^30^ We need to be thoughtful.^31^

## NO: TO A CLINICALLY SIGNIFICANT DEGREE TWINS DO NOT CONSTITUTE AN ADVERSE OUTCOME IN IVF

The preceding section already outlined five of the main arguments of the con-eSET position, all leading to the conclusion that twin deliveries do not constitute an adverse outcome in association with IVF and, indeed, on some occasions should be considered a desired outcome. Since the pro-eSET position, *a priori*, accepted con-eSET’s positions (1) and (2), as already outlined above, they require no further exploration. The same also applies to con-eSET’s position (3), since the pro-eSET argument concurred that, with current knowledge, the conclusion that DETs produce higher clinical and live birth rates than eSETs was unassailable.

Remarkable confluence between pro- and con-eSET positions is also apparent in that the con-eSET argument (4) fully concurs with the pro-eSET position that cost advantages for two consecutive eSETs or DET have not yet been adequately defined. So far published studies on this subject, whether modeled or data-based, have to be viewed as insufficient since they only considered treatment costs, without any considerations given to lifelong earning potentials of newborns. Appropriately conducted cost-effectiveness studies, like any corporate balance sheet, of course, have to consider both sides of the ledger.

A good example for the need of careful assessments of societal costs in association with ART was offered by the largest Canadian province, Quebec, which in August of 2010 agreed to initiate government-paid insurance coverage for three IVF cycles in return for a commitment by the professional community of IVF providers to restrict IVF practice almost exclusively to eSET. As expected, twin pregnancy rates, indeed, precipitously dropped, an effect widely celebrated as success by the medical community.^3^ Yet, as noted in a published retort by us,^32^ this “success” came at considerable cost: the provincial clinical IVF pregnancy rate dropped by 26.2%. In addition, considering lost twin births, the province lost 33.1% of its potential live births from IVF, and their potential lifelong earnings power. By 2015 the province terminated the agreement as “economically unaffordable.”

A similar agreement between government and provider community, offering reimbursement of laboratory expenses for six IVF cycles in return for restrictions on number of embryos transferred, has for many years also been in place in Belgium,^33^ a country with a highly sophisticated IVF industry, yet somewhat disappointing pregnancy and live birth rates (as expressed per cycle initiation).^34^ Similarly, Australia and New Zealand, both countries with highly sophisticated IVF cultures, have been experiencing steady declines in IVF cycle outcomes in parallel with increasing utilization of eSET.^35^ Likely the most drastic negative effects of a broadly implemented eSET policy are, however, observed over the last 10 years in Japan, where national live birth rates over the decade dropped by two-thirds, while IVF cycle starts in the same time period tripled. Current live birth rates per cycle start are around 5.0%.^35^ In order to maintain the number of live births, the country, after 10 years, thus, ended up performing three times as many cycles as before, still only reaching a quite poor live birth rate of approximately 15.0%.

All of these data, of course, also strongly support con-eSET position (4), since they reinforce additionally required efforts from patients and health care providers in compensation of increasing utilization of eSETs.

The most profound differences between pro- and con-eSET positions are apparent in regard to con-eSET position (5): Acknowledged as correct by the pro-eSET position, Gleicher et al. in their publications repeatedly criticized the indiscriminate support of eSET because much of the supportive argument was based on risk extractions derived from retrospective obstetrical data analyses, comparing outcomes in twin (2 offspring) and singleton (1 offspring) cycles. An infertility paradigm, however, is prospective and has to achieve similar outcomes (i.e. 2 offspring). Correct risk comparisons, therefore, have to compare outcomes of one twin and two consecutive singleton pregnancies.^10^–^14^,^32^

The pro-eSET opinion described two studies in the literature, which, indeed, did such appropriate comparisons of one twin and two consecutive singleton pregnancies (all other studies in the literature followed an inappropriate retrospective paradigm, comparing one twin to one singleton pregnancy): both claimed that twin gestations carried higher maternal and neonatal risks.^26^,^27^ A third such study, actually the first ever performed, by Renee Frydman’s group in Paris, was unable to demonstrate such outcome differences but was never published (Lamazou F, Archour-Frydman N, Arbo E, Faivre E, Bourrier MC, Fanchin R, Gomel V, Frydman R. Obtaining two children with IVF: a comparison between one twin and two consecutive single pregnancies. Personal communication, Paris 2011).

Aside from previously noted obvious doubts about the assumption that accomplishment of a singleton pregnancy in an infertile couple can always be repeated, more detailed reviews of these two studies also raise serious questions about their claims. The Swedish paper by Sazonova et al.,^26^ correctly described before in the pro-eSET opinion, indeed, concluded that their study demonstrated *dramatically* higher maternal and neonatal risks following one twin than, summarily, two consecutive singleton pregnancies. This is, however, not what reported data really demonstrate. Specifically, maternal risks for twin pregnancies were, indeed, increased for Cesarean section (OR 4.19, 95% CI 3.32–5.29), PPROM (OR 8.43, 95% CI 4.86–14.63), and pre-eclampsia (OR 2.64, 95% CI 1.81–3.86) but decreased for placenta previa (OR 0.37, 95% CI 0.17–0.81), and there were no differences observed for gestational diabetes and maternal mortality.

Similarly, neonatal risks were higher for sepsis (OR 2.31, 95% CI 1.29 to 4.13), respiratory complications (OR 4.92, 95% CI 3.68–6.58), and jaundice (OR 5.03, 95% CI 3.77–6.70); yet, perinatal mortality, Apgar score <7, first-year mortality, and congenital anomalies did not differ.^26^

What these data, therefore, really demonstrate is that two consecutive singleton pregnancies, indeed, demonstrated mildly lower maternal and neonatal risks than a single twin pregnancy; but to describe these increased risks as *dramatic*, as the authors did, does not appear warranted. In the absence of effects on neonatal Apgar scores below 7, any significant clinical impact on neonates is highly unlikely and, therefore, not compensatory for undisputed losses in pregnancy and live birth chances as well as increased efforts.

This conclusion is also supported by similar results, reported by La Sala et al.^27^ Though these authors also concluded that twin deliveries cause more complications than two consecutive singletons, it is remarkable that they, too, demonstrated absolutely no increase in intrauterine fetal demise, neonatal death, perinatal mortality, and even NICU admissions. One therefore has to conclude from both of these studies that, if twins indeed represent increased risks, at worst they are too mild seriously to affect neonatal outcomes.

It has been known for over a decade that spontaneously conceived pregnancies have very different risk profiles from pregnancies conceived through IVF.^18^ The risk discussion, therefore, does not end here. Already in 2004 the Dutch investigators Helmerhorst et al. reported after review of the then existing literature on the subject that, while singleton IVF pregnancies carry higher risks than spontaneously conceived singletons, twin IVF pregnancies have an approximately 40% lower risk profile than spontaneously conceived twins.^18^ We recently updated those authors’ review, demonstrating that, despite overall improvements in obstetrical outcomes, more recently published data still almost exactly mirrored the outcome differences initially reported by the Dutch investigators. Exaggerations of severe risks in IVF twin pregnancies in comparison to spontaneously conceived were in the 50% range, while exaggerations of milder perinatal risks were found in the approximately 25% range.^14^

Combining these highly significant findings with, at best, marginally increased risks noted in IVF patients with twins over two consecutive singletons, one is left with the conclusion that twin deliveries, with considerable likelihood, do not increase outcome risks to clinically significant degrees and maybe even result in an overall lower risk profile for mothers and offspring. However, even under worst-case assumptions of a mildly increased risk profile for twins, one still has to wonder whether that would warrant certain declines in live birth chances and, therefore, additional efforts to catch up.

## CONCLUSIONS FROM PRO- AND CON-ESET OPINIONS

Assuming this to be the case, and in full appreciation of the previously noted twin birth rate high of 33.9 per 1,000 live births, one has to wonder about the concentration on IVF-generated twins by the medical profession, insurance companies, and government agencies, when IVF-generated twins represent only 15.7% of the national twin birth rate.^23^ With prevention of naturally conceived twins impossible, and IVF contributing so little, it appears time to concentrate on the primary culprit in not only a large majority of fertility treatment-related twin pregnancies but also in higher-order multiple pregnancies. Non-IVF fertility treatments, often still conducted in general obstetrical and gynecological offices, have been identified as the main cause of all multiple births for quite some time.^29^–^31^,^36^–^38^ Yet, paradoxically, as here extensively discussed, educational and regulatory efforts over the last decade have only been directed at the practice of IVF.

Considering that IVF patient populations in the US and other developed countries are rapidly aging and, as noted before, any argument against taking chances with twin deliveries becomes increasingly difficult to make with advancing female age and/or declining ovarian reserve, it is difficult to understand why non-IVF fertility treatments, usually applied to younger women, have so far escaped attention in discussing the high twin birth rate in the United States. In offering this debate on eSET utilization in association with IVF, we hope to have contributed to a redirection of priorities.

## Abbreviations

ART: assisted reproductive technology
ASRM: American Society for Reproductive Medicine
DET: double embryo transfer
eSET: elective single embryo transfer
IVF: *in vitro* fertilization
NICU: neonatal intensive care unit
PPROM: preterm premature rupture of membranes
